# A new method for identifying and mapping areas vulnerable to Covid-19 in an armed conflict zone: Case study north-west Syria

**DOI:** 10.1016/j.mex.2020.101091

**Published:** 2020-10-09

**Authors:** Samira Mobaied

**Affiliations:** SU - Sorbonne Université IRD, Patrimoines locaux, environnement et globalisation (PALOC, UMR 208), Muséum national d'Histoire naturelle, Case Postale 26, 57 rue Cuvier, 75231 Paris Cedex 05, France

**Keywords:** Covid-19, Syria, Risk map, Information system (GIS), Spatial model

## Abstract

In the context of the Covid-19 pandemic, a method for identifying and mapping vulnerable areas in an armed conflict zone seems necessary to limit the risk and anticipate the spread of contamination. It may also assist in the preparation of health infrastructures and the development of strategies to manage such infrastructures as this pandemic, which affects the whole world and has created chaos in fragile states, is causing significant problems in armed conflict zones. To achieve these objectives, geographic information technologies, remote sensing and spatial modelling currently offer new potential for anticipating the spread of risk in armed conflict zones and better managing health or natural emergencies.

In this paper, we present the Risk of Vulnerability to COVID-19 in War Zones Index “Id_Covid19_WZ”. This index was calculated based on several factors and by using spatial data. A risk map was then created from this data developed for the north-west of Syria, an area where there has been intense fighting for several years.

• Identify areas vulnerable to the Covid-19 pandemic.
• Anticipating the spread of risk in armed conflict zones.
• Using remote sensing and spatial modelling to managing health emergencies.

## Graphical abstract

**Figure:**
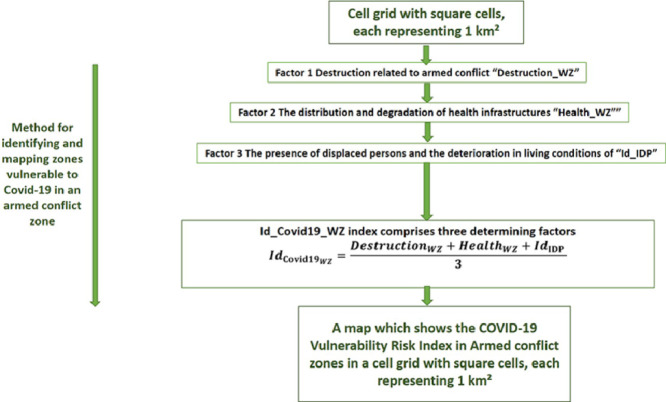

Specifications Table

**Table:** 

Subject Area	Engineering
More specific subject area	Use of GIS in Crisis management
Method name	Mapping zones vulnerable to Covid-19
Name and reference of original method	Method was developed as a way to identify and map zones vulnerable to pandemics, no primary reference is available.
Resource availability	All metadata and methods are available as a dataset published in the paper.

## Method details

### Description of the Id_Covid19_WZ index

The Risk of Vulnerability to Covid-19 in War Zones Index “Id_Covid19_WZ index” aims to identify areas vulnerable to the Covid-19 pandemic [4,9], in an armed conflict zone to help decision-makers limit the risk and anticipate the spread of contamination.

The Id_Covid19_WZ index comprises three determining factors (Tables 1 and2):

$$Id_{\text{Covid}19_{\text{WZ}}}=\frac{\text{Destructio}n_{\text{WZ}}+\text{Healt}h_{\text{WZ}}+Id_{\text{IDP}}}{3}$$

**Table 1:** List of abbreviations and acronyms used in this article.

Abbreviation	Explanation
WZ	War Zones
Destruction_WZ	Destruction related to armed conflict
Bomb	Proximity to bomb sites
Fatality	Bombardment mortality rate
Health_WZ	Distribution and degradation of health infrastructures
Prox_Health	Proximity of health facilities
Fonc_Health	Operational status of health care facilities
Id_IDP	Presence of displaced persons and deterioration of living conditions
Prox_ Camp	Proximity of refugee camps
nbr_IDP	Number of displaced persons

**Table 2:** Intermediate indices values.

Indices	Values
Proximity of bomb sites (Km)	Value “Bomb” index
0.5	1
1	0.75
1.5	0.5
>1.5	0.1
Mortality rate	Value “Fatality” index
0 > 10	1
5 > 10	0.75
1 > 5	0.5
1	0.1
Proximity of health facilities (Km)	Value “Prox_Health” index
1	0.1
3	0.5
5	0.75
>5	1
Functional capacity of healthcare facilities	Value “Fonc_Health” index
Fully functioning hospital	0.1
Fully functioning health centres	0.5
Partially functioning health centres	0.75
No health facility within a radius of 5 km	1
Proximity of refugee camps (Km)	Value “Prox_ Camp” index
> 1	1
2	0.75
4	0.5
>4	0.1
Number of displaced persons “nbr_IDP”	Value “nbr_IDP” index
120,000	1
90,000	0.75
60,000	0.5
<25,000	0.1

#### Factor 1 destruction related to armed conflict “Destruction_WZ”

Calculation of this factor is based on two essential elements: the proximity of the bomb sites, “Bomb”, and the intensity of the bombing, “Fatality”. The proximity of the bomb sites has been calculated by assigning a value [0–1] based on the distance from the bomb sites. The value of the “Bomb” index is lower if there is no combat in the vicinity (within a radius of 1.5 km) and this value increases progressively up to the value 1 if it is a site that has been bombed during the last six months. The bombardment mortality rate, “Fatality”, was calculated as an indicator of the intensity of the attacks by assigning a value [0–1] based on the mortality rate according to data provided in the ACLED database [1]. The “Fatality” index has a low value when the mortality rate at the site is low and this value increases as the mortality rate increases.

$$\text{Destructio}n_{\text{WZ}}=\frac{\text{Bomb}+\text{Fatality}}{2}$$

#### Factor 2 the distribution and degradation of health infrastructures “Health_WZ”

Calculation of this factor is based on two essential elements: the proximity of health facilities and the state of operation of health care institutions. The proximity of health facilities, “Prox_Health”, was calculated by assigning a value [0–1] based on the distance from a health facility. The value is lower if there is a health facility within a radius of 1 km and this value increases progressively up to the value 1 if no health facility is present within a radius of 5 km. The operational status of health care facilities was calculated by assigning a value [0–1] based on the operational status of the health care facilities: this “Fonc_Health” index takes the maximum value of 1 when the health care facility is out of service. The value decreases as the functional capacity of the health care facility increases.

$$\text{Health}\_\text{WZ}=\frac{\text{Prox}\_\text{Health}\;+\text{Fonc}\_\text{Health}\;}{2}$$

#### Factor 3 the presence of displaced persons and the deterioration in living conditions of “Id_IDP” (internally displaced people)

Calculation of this factor is based on two essential elements: the presence of refugee camps and the number of displaced persons. The proximity of refugee camps “Prox_ Camp” was calculated by assigning a value [0–1] based on the distance from a refugee camp. The value of this index is 1 if a refugee camp is present within a radius of 1 km from the site, the value decreases to 0.1 if no refugee camp is present within a radius of 4 km.

The number of displaced persons, “nbr_IDP”, has been calculated by assigning a value [0–1] which is the maximum value of 1 in areas where the number of IDPs exceeds 120,000 persons and gradually decreases to 0.1 in areas where the number of IDPs does not exceed 25,000 persons.

$$\text{Id}\_\text{IDP}\;=\frac{\text{Prox}\_\;\text{Camp}\;+\text{nbr}\_\text{IDP}\;}{2}$$

## Data processing

The following data was used to calculate the “Id_Covid19_WZ” index:

Data on health care facilities and their status in north-western Syria was extracted from Syria's health sector bulletin, developed by the World Health Organisation, together with the functional status of each facility [6], [7], [8].

Data on the bombings that took place in this zone over the previous six months was extracted from the Armed Conflict Location and Event Data Project (ACLED) website, as was the fatality rate of each attack [1].

The data produced on refugee camps and the number of displaced persons in these zones was extracted from maps prepared by the United Nations Office for the Coordination of Humanitarian Affairs [2,3].

All of the data was extracted for a zone of about 7200 km² in north-western Syria, a region with a population of about 3.5 million inhabitants, most of whom are displaced persons and refugees, where intensive fighting has been going on for several years.

The input data was integrated into the cell grid using numerical methods with square cells each representing 1 km². From this data, the value of each index was calculated in each cell by applying the formulae described in this paper using spatial calculation methods. Data analysis and processing was carried out using ArcMap/ArcGIS Desktop GIS software version 10.5 [5].

## Value of the Data

• This paper presents a spatial index “Id_Covid19_WZ” that allows maps of areas vulnerable to Covid-19 in an armed conflict zone to be developed. This method can be used in any conflict zone in the world.
• This paper presents a map and a table of the “Id_Covid19_WZ” index values, which show the areas vulnerable to Covid-19 in a combat zone in the north-west of Syria. In this zone, the index was calculated and mapped with a resolution of 1 km².
• The database resulting from this paper can be used to manage the pandemic in this region by preparing the most vulnerable zones with the necessary health facilities and protective measures.

## Data Description

**Fig. 1:**
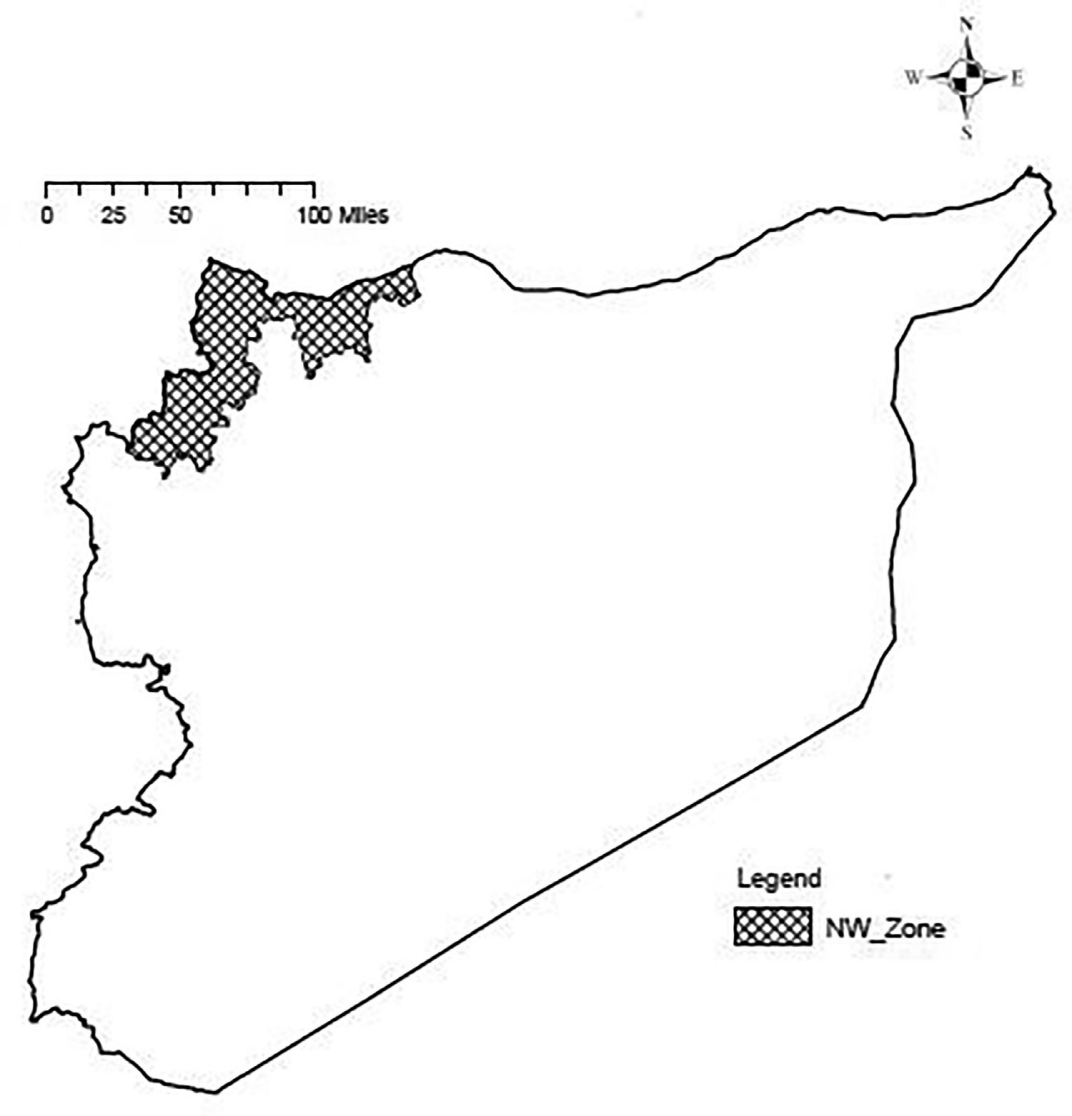
A map of Syria showing the combat zone in the north-west of Syria.

The Covid-19 vulnerability index in an armed conflict zone “Id_Covid19_WZ” was calculated for a 7703-cell grid with square cells each representing 1 km², giving a total grid size of 7703 km² (Fig. 1).

The Id_Covid19_WZ index varies between [0–1]. Its value is zero when the risk of propagation of Covid-19 is minimal and it increases in relation to the increasing vulnerability of zones to the spread of the Covid-19 virus up to the value of 1, which indicates maximum risk.

Table: a table containing 7703 records representing the value of Id_Covid19_WZ included in this paper with the spatial coordinates of each cell under the GCS_WGS_1984 geographic coordinate system.

The results of the calculation of the vulnerability risk index for Covid19 in North-West Syria are presented in two forms:

1. A map of north-west Syria showing the Covid-19 Vulnerability Risk Index in Armed Conflict Zones (Fig. 2). Available at: https://qgiscloud.com/esmobaied/Grille_Covid19_NWS_25062020/.

**Fig. 2:**
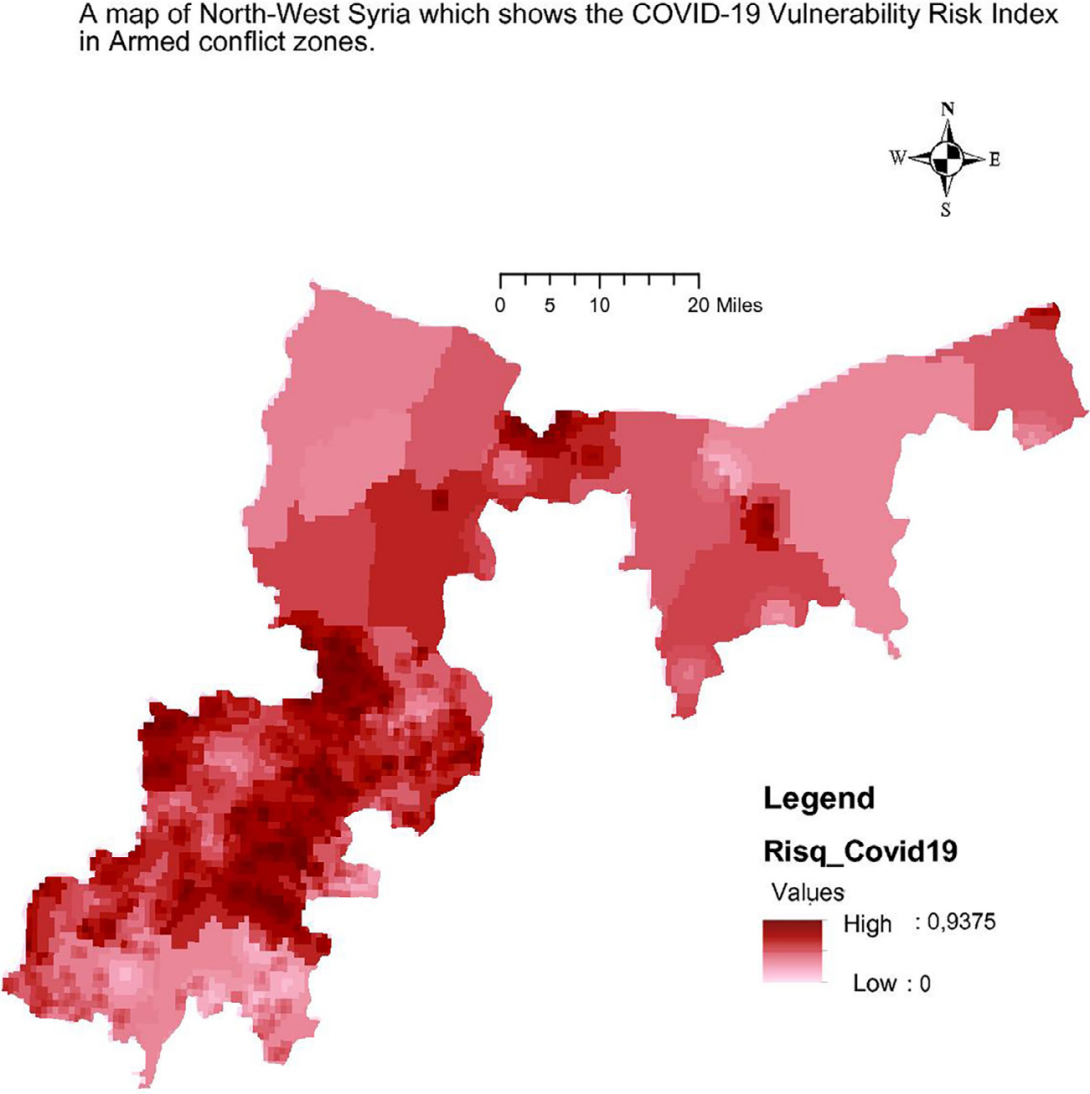
A map of 7703 km² of north-west Syria included in this paper, which shows the Covid-19 Vulnerability Risk Index in Armed conflict zones in a cell grid with square cells, each representing 1 km².
2. A table containing 7703 records representing the value of Id_Covid19_WZ in a cell grid with square cells, each representing 1 km² with the spatial coordinates of each cell under the GCS_WGS_1984 geographic coordinate system.

## Results

The Covid19 vulnerability index in an armed conflict zone “Id_Covid19_WZ” was calculated for a 7703-cell grid with square cells each representing 1 km², giving a total grid size of 7703 km².

The Id_Covid19_WZ index varies between [0–1]. Its value is zero when there is minimal risk of Covid-19 propagation and it increases with the increasing vulnerability of zones to the spread of the Covid-19 virus up to the value of 1, which indicates maximum risk.

This paper presents a spatial index “Id_Covid19_WZ” that allows maps of areas vulnerable to Covid-19 in an armed conflict zone to be developed. This method can be used in any conflict zone in the world.

## Conclusion

This paper presents a new method for identifying and mapping vulnerable areas. This method can be used in any conflict zone in the world. The map resulting from this paper can be used to manage the pandemic in this region by preparing the most vulnerable zones with the necessary health facilities and protective measures.

## Declaration of Competing Interest

The authors declare that they have no known competing financial interests or personal relationships that have, or could be perceived to have, influenced the work reported in this article.

## Appendix Supplementary materials

Image, application 1
